# F141. DUTY TO WARN FOR POTENTIAL RISK OF PSYCHOLOGICAL HARM: A CASE REPORT

**DOI:** 10.1093/schbul/sby017.672

**Published:** 2018-04-01

**Authors:** Naista Zhand, David Attwood

**Affiliations:** University of Ottawa Faculty of Medicine

## Abstract

**Background:**

Under the current ethical and legal standards physicians are expected to breach confidentiality when a third party is at risk. However, the law has mainly focused on risk of bodily harm and there is no legal guidance on physicians’ responsibilty when it comes to risk of psychological harm to a potential targeted victim

**Methods:**

This case report illustrates a clinical dilemma on duty to warn for mental health professionals.

**Results:**

N/A

**Discussion:**

This case report illustrates an unconventional perspective on a psychiatrist’s duty to warn: consideration of risk of psychological harm to the potential target of their patient. Psychological trauma can have a potential negative impact on victims, affecting their mental health and well being as well as their physical health. The ethicolegal dilemma discussed in this case has implications for policies related to the care of psychiatric patients.

